# Osteoclast-like Cells in Early Zebrafish Embryos

**Published:** 2014-05-25

**Authors:** Faiza Sharif, Merijn A.G. de Bakker, Michael K Richardson

**Affiliations:** 1 Interdisciplinary Research Center in Biomedical Materials, COMSATS Institute of Information Technology, Defence Road, Off Raiwind Road, Lahore, Pakistan; 2 Institue of Biology, Leiden University, Sylvius Laboratory, Leiden, The Netherlands

**Keywords:** Zebrafish, Osteoclasts, TRAcP, *Ctsk*, *mmp-9*

## Abstract

**Objective:**

Genes involved in bone and tissue remodelling in the vertebrates include *matrix metalloproteinase-9* (*mmp-9*), receptor activator of necrosis factor κ-β (*rank*), *cathepsin-k* (*Ctsk*) and tartrate-resistant acid phosphatase (TRAcP). We examine whether these markers are expressed in cells of zebrafish embryos of 1-5 days post fertilization. We also examine adult scales, which are known to contain mature osteoclasts, for comparison.

**Materials and Methods:**

In this experimental study, *in situ* hybrdisation, histochemistry and serial plastic and paraffin sectioning were used to analyse marker expression.

**Results:**

We found that *mmp-9* mRNA, TRAcP enzyme and *Ctsk* YFP protein were expressed in haematopoietic tissues and in the cells scattered sparsely in the embryo. *Ctsk* and *rank* mRNA were both expressed in the branchial skeleton and in the developing pectoral fin. In these skeletal structures, histology showed that the expressing cells were located around the developing cartilage elements, in the parachondral tissue. In a transgenic zebrafish line with YFP coupled to *Ctsk* promoter, *Ctsk* expressing cells were found around pharyngeal skeletal elements. To see whether we could activate osteoclasts, we exposed *prim-6* zebrafish embryos to a mixture of 1 µM dexamethasone and 1 µM vitaminutes D3. These compounds, which are known to trigger osteoclastogenensis in cell cultures, lead to an increase in intensity of *Ctsk* YFP expression around the skeletal elements.

**Conclusion:**

Our findings show that cells expressing a range of osteoclast markers are present in early larvae and can be activated by the addition of osteoclastogenic compounds.

## Introduction

We present our original research where we examine the expression of genes involved with bone and tissue remodelling in zebrafish larvae. Bone and tissue remodelling are normal processes in adult animals, and in developing embryos. There are two different kinds of cells associated with bone remodelling, namely osteoclasts and osteoblasts which work in harmony in a normal healthy system (1). Any irregularity or abnormality in the remodelling process leads to certain pathological conditions such as osteoporosis (2).

In some previous studies, mature osteoclasts have only been detected in developing zebrafish after 20 days post fertilization (dpf). These cells are initially mononucleated, although multinucleated osteoclasts are also present in the adult zebrafish (3). A recent review (4) has shown that the bone of zebrafish is osteocytic or cellular, similar to mammalian models. Furthermore, those authors concluded that the osteocytes of zebrafish are mesenchymal cells, analogues of the osteoblasts of mammals. The multinucleated osteoclasts of zebrafish display similar features to that of mammalian multinucleated osteoclasts, for example formation of Howship’s lacunae and staining positive for tartarate resistant acid phosphatase (TRAcP) enzyme (3).

Resorption by large multinucleated cells is lacunar, whereas resporption by mononucleated cells is shallow and non-lacunar in many teleost species (5, 6). Therefore, evidence from humans and other mammalians as well as teleosts suggests that a significant number of active osteoclasts are mononucleated (4, 5) . In addition to a number of similarities with human and mammalian bone resorption, there are differences in the regulation of mammalian to fish osteoclasts. The site of osteoclast origin in teleost fish is not the bone marrow (7) ; therefore, it is considered that osteoclasts are formed in the head, kidney and spleen (8, 9).

Two molecules which are essential for initiating osteoclastogenesis are macrophage colonystimulating factor (M-CSF) and receptor for activation of nuclear factor kappa B (NF-κB) *RANK* ligand (*RANK*-L). Both of these are expressed by stromal cells related to osteoblasts. *RANK* is itself expressed on osteoclast precursors (10). *Ctsk* and *matrix metalloproteinase-9* (*MMP-9*) are expressed strongly in multinucleated osteoclasts and weakly in pre-osteoclasts (11).

Another important enzyme in this context is tartarate resistant acid phosphatase (TRAcP) which is expressed in activated murine and teleost osteoclasts (3). TRAcP is involved in hydrolysis of various substrates including components of bone matrix. The mono- and multinucleated osteoclasts of teleosts secrete TRAcP at the site of active bone resorption (6, 12-14). Thus, TRAcP-staining can be specific for osteoclastic bone resorption (and also for sites where osteoclasts were previously active) (3, 5, 14). Ballanti et al. (15) regard expression of this marker as one of the best ways to identify osteoclastic cells.

*RANK*, together with its ligand, the TNF-family molecule *RANK-L* (TRANCE, or osteoclast differentiation factor, ODF), is a key regulator of bone remodelling, and is essential for the development and activation of osteoclasts (16). *RANK* is expressed by osteoclast progenitors, mature osteoclasts, chondrocytes and mammary gland epithelial cells (17, 18). Moreover, hormones or cytokines that stimulate bone resorption such as 1,25-dihydroxy vitamin D3 [1,25(OH)2D3, also called vitamin D3], parathyroid hormone (PTH), members of the interleukin (IL)-6 family, or IL-1 stimulate osteoclast formation by activating discrete signaling pathways in stromal /osteoblastic cells (19). Non adherent marrow mononuclear cells were activated by the addition of vitamin D3 in cultures (20, 21). It has been found in an earlier work that dexamethasone enhanced osteoclast-like cell formation induced by 1, 25-(OH) 2D3 in murine bone marrow cell cultures (22) .

*Ctsk* as a cysteine protease expressed by osteoclasts and synovial fibroblasts is responsible for removing the organic matrix, mainly fibrilar type- 1 collagen, and for solubilisation of the inorganic component (hydroxyapatite). Similarly, members of the matrix metalloproteinase (MMP) family of genes are important for the remodelling of the extracellular matrix (ECM) in a number of normal biological processes (23). They also perform the same function, i.e. ECM remodelling in some pathological processes, including cancer metastasis, and rheumatoid arthritis. Finally, MMPs are present in some snake venoms, where they may be involved in lysis of tissue in the prey (24, 25).

It is important to understand the processes underlying the remodelling, since disease can arise if these are dysregulated. Therefore, as a step towards establishing a zebrafish model for bone diseases, we have characterized the expression of panel of osteoclast-associated markers in early stages of zebrafish development. Early stages are particularly desirable for establishing a model because they are free from the legal restrictions that apply to the use of adults, and are, therefore, more suited to high throughput assays. To establish the fact that the cells observed were of osteoclastic origin, it was important to see if these cells could be activated by some osteoclastogenic compounds such as dexamethasone and vitamin D3. It is much easier and more effective to see in the transgenic embryos expressing osteoclast markers for example newly developed transgenic *cathepsin-k* zebrafish embryos.

## Materials and Methods

### Animals

In this experimental study, Danio rerio (zebrafish) was used as the animal model for expression profiling of selected genes and proteins. All experimental procedures were conducted in accordance with the Netherlands Experiments on Animals Act that serves as the implementation of "Guidelines on the protection of experimental animals" by the Council of Europe (1986), Directive 86/609/EC, and were performed only after a positive recommendation of the

Animal Experiments Committee had been issued to the licensee. Spawning of Danio rerio took place at 26˚C in aerated 5 litre tanks, in a 10 hours: 14 hours light: dark cycle. In each mating setup, two females and one male fish were placed together. The eggs are usually laid after first light in the morning. They were collected within the first hour, sorted and distributed in Petri dishes, filled with egg water (60 μg/ml of instant ocean salt).

The eggs were cleaned and transferred to 9 cm Petri dishes at a concentration of 60 eggs per dish. They were maintained at 28˚C in atmospheric air in a climate cell, also with a 10 hours: 14 hours light: dark cycle. The Petri dishes were checked after 4 hours and 8 hours for dead and unfertilized eggs, respectively, which were removed and discarded. After continued incubation in the Petri dishes at 28˚C, embryos were harvested each morning from day 1 to day 5 and fixed as follows. Eggs were immersed in 4% buffered paraformaldehyde (PFA) at 4˚C overnight, dehydrated in an ascending series of methanol staring from 25 to 100%, and finally, stored at -20˚C in 100% methanol. One- and two-day old embryos were dechorionated before fixation.

### Cloning of genes and synthesis of probe

The NCBI genbank was searched for homologous sequences with the Blast X algorithm using zebrafish query. Only for *Ctsk* (Goldfish, *Carassius auratus*, AB236968) and *MMP-9* (Common carp, *Cyprinus carpio*, AB057407) did we find similar enough sequences for our goal and aligned them with their zebrafish counter parts. These alignments were used to design polymerase chain reaction (PCR) primers based on conserved regions. The other primers were designed only using, mostly multiple, zebrafish sequences. Primers are shown in table 1.

**Table 1:** Nucleotide sequence of primers used for PCR


Primer	Nucleotide sequence 5`-3`	Position

**Receptor activator of necrosis factor Kappa B**		
**RANK F1**	TGGCGGAAGGAAAGATTCCTC	157
**RANK F2**	TGTGGCTCTGACCGCAGTCC	1071
**RANK R1**	CGCAGTCCGGCTGACTCTG	1060
**RANK R2**	CTGGGACTTTGCTGCAGTAGATGC	243
**Cathepsin-K**		
**CTS K F1**	GATGAGGCTTGGGAGAGCTGGAA	180
**CTS K F2**	GACGATTTGGGAGAAGAACATGCTG	256
**CTS K R1**	TTTCGGTTACGAGCCATCAGGAC	1013
**CTS K R2**	CCCTTCTTTCCCCACTCTTCACC	1063
**Matrix metalloproteinase 9**		
**MMP 9 F1**	TTCGTGACGTTTCCTGGAGATGTG	207
**MMP 9 F2**	CACAGCTAGCGGATGAGTATCTGAAGC	253
**MMP 9 R1**	TGGCTCTCCTTCTGAGTTTCCACC	1120
**MMP 9 R2**	AATGGAAAATGGCATGGCTCTCC	1135


The PCR parameters consisted of 5 minutes of denaturation, followed by 40 cycles of denaturation at 95˚C for 10 seconds, annealing for 10 seconds, and extension for 60 seconds ending with 10 minutes of extension at 72oC. The PCR products chosen for cloning had the following primer combinations: CTSK F1 & R2, *MMP-9* F2 & R2 and *RANK* F2 & R2. The PCR products were cleaned using Wizard SV Gel and PCR Cleanup system (Promega: Leiden, the Netherlands).

The ligation, cloning and transformation of plasmids in competent cells were done with the TOPOTA PCRII kit from Invitrogen (Breda, the Netherlands). White colonies of transformed cells were grown and checked by PCR. Samples were then sent for sequencing to Macrogen, USA. All the sequence analysis showed a strong homology with the reference sequences. Linearization of template was done with restriction enzymes XbaI, XhoI, HindIII or BamHI, depending on the direction of the gene and potential restriction sites present in the product, and cleaned with Wizard SV Promega columns (Promega, USA). T7 or SP6 RNA-polymerases (Roche, Germany) were used to synthesis the digoxigenin labelled RNA probes. The probes were stored at -20˚C. Also, sense probes were prepared for all of the genes, and used as negative control in *in situ* hybridization (ISH). The genbank accession numbers of our PCR products are shown in table 2.

**Table 2:** Genbank accession numbers of the genes cloned and used for *in situ* hybridization in this study


Gene	Genbank accession number

***MMP-9***	HM239640
**CTS-K **	HM239643
**CTS-K-1b **	HM239644
**Rank **	HM239645
**TRACP**	HM239646


### In situ hybridization (ISH)

Fixed and dehydrated embryos (see above) were rehydrated from methanol by passing through descending concentration of methanol, namely 75, 50 and 25%. They were then washed twice in 1x phosphate buffered saline (PBS) with 0.2% Tween 20 (PBST) for 10 minutes each. In some cases, embryos of 2-5 dpf were then bleached at room temperature with hydrogen peroxide until pigmentation had completely disappeared (10 to 12 minutes). One-day old embryos were found to be too delicate for bleaching. Then, they were washed twice in PBST for 5 minutes. Embryos were treated with Proteinase-K (10 μmg/ml) for incubation time that was varied according to the following stages: 1 dpf for 10 minutes, 2 dpf for 15 minutes, 3 dpf for 20 minutes, and 4 and 5 for 40 minutes. Further processing of embryos was conducted, with minor modifications, according to Xu Q and D.G.Wilkinson (26).

### Adult fish scales

We fixated adult zebrafish skin with scales in 4% PFA at 4˚C overnight and stored them in 100% methanol after dehydration. In situ hybridization was carried out as described above for embryos except that no bleaching was done, while proteinase-k treatment was done for 10 minutes.

### Transgenic *Ctsk* larvae

*Ctsk* transgenic YFP labelled zebrafish published recently (27) were kindly provided by Prof. Stefan Schulte-Merker. The eggs were obtained from one pair of these transgenic fish and were cleaned and sorted as mentioned above [see ‘Animals’ At the *prim-6* stage (28)]. The transgenic embryos were continuously exposed to a mixture of 1 μM dexamethasone (DEX) (Sigma Aldrich, Switzerland) and 1 μM vitaminutesD3 (1, 25-dihydroxyvitaminutes D3) (cholecalciferol; SERVA electrophoresis GmbH, Germany) for 4 days. Controls were treated in the same way except that DEX and vitaminutes D3 were not added. At 5 dpf, control and treated larvae were observed alive under a confocal microscope (Zeiss Observer, CLSM, Germany) and images were taken for measurement of fluorescence intensity. The same conditions and exposure settings were used for all images. The fluorescence intensity of controls and treated larvae was measured using image J software (National Institute of Health, USA). The number of embryos analysed ranged between 10 and 12. Unpaired t-test was done for statistical analysis of fluorescence intensity.

### TRAcP enzyme staining

Normal zebrafish embryos and adult scales were preserved in 100% methanol after rehydration through a graded series of methanol in PBST. TRAP enzyme kit (Sigma-Aldrich 387A- 1KT, Chemie GmbH, Steinheim, Germany) was used for staining the embryos and scales according to the user instructions.

### Sectioning

Sections were prepared from the whole mount ISH larvae. The larvae were dehydrated in graded ethanol, and embedded in wax using Histoclear (Thermo Scientific, USA) as the intermediate reagent. Some sections were prepared with Technovit (Technovit, Kulzer Heraus, Germany) embedding after dehydration. The thickness of sections was 5 μm.

### Imaging

Imaging of *in situ* embryos and sections was done using a Nikon eclipse E800M (Japan) equipped with a DSF1 camera. For *in vivo* confocal imaging of the transgenic larvae, they were immobilized in low melting agarose (1%) after anesthesia (0.04% Tricaine, Finquel, USA)). Imaging was done using a Zeiss observer LSM 500 inverted microscope (Carl Zeiss BV, Sliedrecht, The Netherlands).

## Results

In situ hybridization results for *Ctsk*, *rank* and *mmp-9* are shown in figs 1-9. Expression patterns of the genes are summarized in table 3.

**Table 3:** Summary of expression patterns of osteoclast markers in zebrafish development found in this study


Marker	Expression

***Ctsk***	pharyngeal skeleton and pectoral fin, and inCHT in case of (transgenic larvae)
**Rank **	pharyngeal skeleton and pectoral fin
***mmp-9***	scattered cells on whole body and CHT
**TRAcP**	scattered cells on whole body and CHT (at5 dpf in Meckel’s cartilage)


### Ctsk

**Fig 1:**
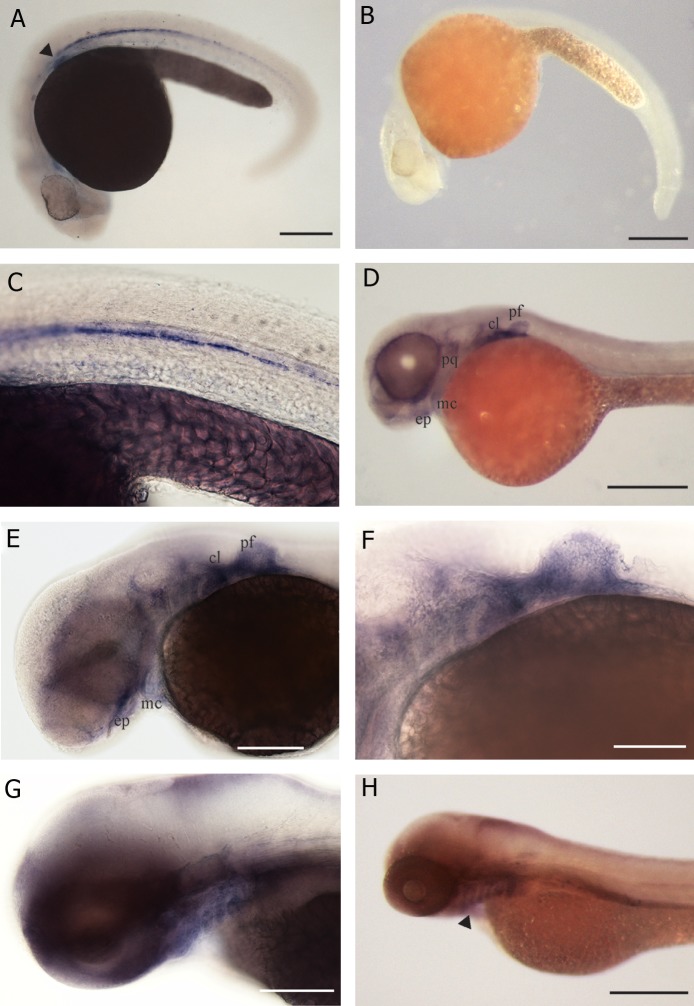
*Ctsk* gene expression by *in situ* hybridization in zebrafish embryos, scale bar=200 μm unless otherwise mentioned. A. Expression in rostral blood island, lateral sides of notochord and pectoral fin bud (arrow head), in 1 dpf embryo. B. Sense control of fig A with no specific expression. C. Detail of fig 1A showing expression in the lateral region adjacent to the notochord, (arrow heads) scale bar=100 μm. D. 2 dpf embryo with expression in the Meckel’s cartilage (mc), ethmoid plate (ep), cleithrum (cl) palatoquadrate (pq) and pectoral fin bud (pf). E. Detail of fig D scale bar=150 μm. F. Detail of fig E showing expression in the pectoral girdle and fin bud, scale bar=50 μm. G. 3 dpf zebrafish embryo showing expression in the pharyngeal arches, scale bar=200 μm. H. 4 dpf larva with expression in the pharyngeal arches (arrow head).

**Fig 2:**
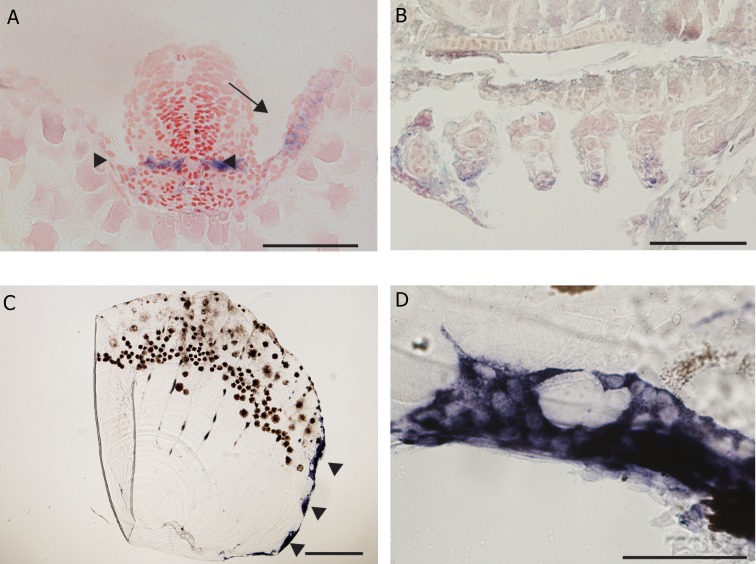
*Ctsk* gene expression in zebrafish larvae and scales by *in situ* hybridization. A. Histological section through the posterior region of 1 dpf embryo expressing *Ctsk* gene in the cells of adaxial mesoderm, (arrow heads) and pectoral fin bud (arrow), scale bar=100 μm. B. Section through pharyngeal arches of 5 dpf larva showing expression around the cartilaginous elements within the arches, scale bar=50 μm. C. Whole mount adult scale showing expression at the margin, scale bar=300 μm (arrow heads). D. Putative multinucleated cell expressing *Ctsk* in the marginal region of a scale, scale bar=20 μm.

Hybridization patterns of *Ctsk* are illustrated in figure 1A-H and figure 2A-D. Embryos hybridized with sense probe showed no specific signal (Fig 1B). With antisense probe (Figes1A, C-H and 2A-D), there was expression as follows. At 1 dpf, hybridization was seen in a longitudinal stripe of adaxial mesoderm cells along each side of the trunk (Fig 1A), the stripe being interrupted at intersomitic boundaries (Fig 1C). Histological sections (Fig 2A) showed that this staining was paranotochordal, extending laterally from the notochord towards the epidermis. Hybridization was also seen in the pectoral fin buds (Fig 1A).

At 2 dpf, specific expression in whole mounts was seen in the skeletal elements in the head, (trabeculae cranii, branchial arches, and lower jaw) and in the cleithrum and pectoral fin buds (Fig 1D, E, F). Histological sections showed that expression was peripheral to the cartilage that is perichondrium, (Fig 2B). By 3 dpf, the expression in the pectoral fin was reduced, and by 4 dpf, the expression was faint (Fig 1G, H). At 5 dpf, expression in the branchial arches was indistinct in whole mounts, but was still visible in histological sections (Fig 2B), where specific expression was again seen in the mesenchymal cells, adjacent to the cartilaginous elements of the branchial arches. However, as with the pectoral fin and the lower jaw, there was no staining in the cartilage itself.

To confirm the specificity of the probe, we examined expression in the adult scales. Here, the hybridization was specific and distinct. Large clusters of heavily-stained cells were localized at the margins of the scales (Fig 2C). At higher magnifications, these clusters could be seen to have many nuclei, the dark staining being localized to the cytoplasm (Fig 2D).

### Rank

**Fig 3:**
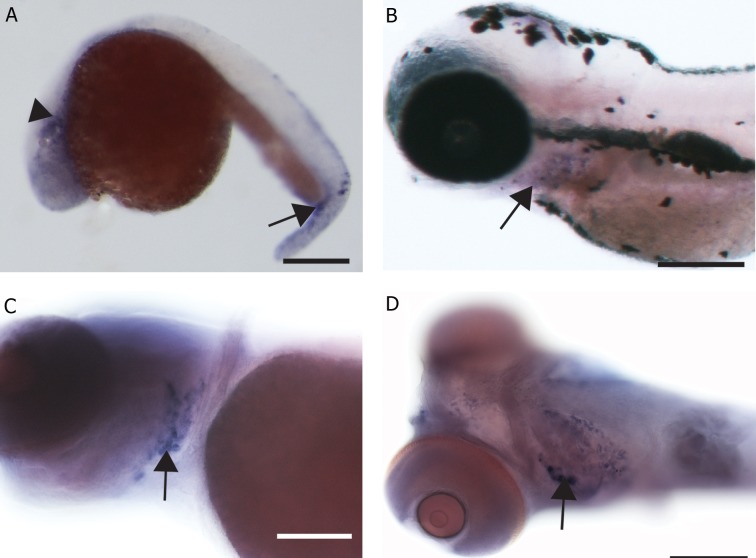
Rank gene expression in zebrafish embryos. A. 1 dpf embryo expressing rank gene in the rostral blood island (arrow head) and ventral blood island (arrow) in addition to staining in the jaw region, scale bar=300 μm. B. 3 dpf larva expressing rank in the pharyngeal arches, (arrow) scale bar=150 μm. C. 4 dpf larva expressing rank in the pharyngeal arches (arrow), scale bar=200 μm. D. Ventral view of 4 dpf larva with expression in the pharyngeal arches (arrow) and anterior end of the Meckel’s cartilage, scale bar=200 μm.

**Fig 4:**
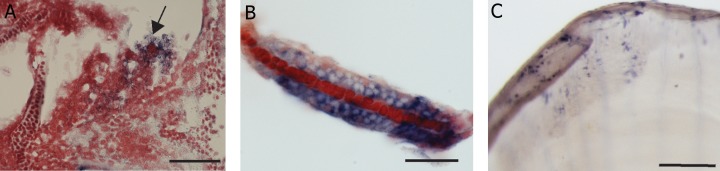
Rank gene expression in zebrafish larvae and scales. A. Histological section through pharyngeal arches showing rank expression in the arches (arrow), scale bar=50 μm. B. Section through pectoral fin of 2 dpf embryo showing rank expression in the tissue around the cartilage (red), scale bar=100 μm. C. Whole mount adult scale with rank expressing cells on the margin, scale bar=100 μm

Hybridization patterns with *rank* antisense (Fig 3A-D) and sense probes (Fig not shown). Sense controls showed no hybridization. At 1 dpf, diffuse expression was observed in the head region, around the yolk sac and in the tail in the region of the ventral blood island (Fig 3A) where *mmp-9* and *Ctsk* expression was also seen. At 2 dpf, expression was observed in the cleithrum and pectoral fin bud. At 3 and 4 dpf, *rank* expression was seen in the pectoral fins and the branchial arches (Fig 3B, C). At 5 dpf, expression was seen only in the branchial arches (Fig 3D). Histological sections through the branchial arches of 5-day old larvae confirmed that expression was in the loose mesenchyme, but not the cartilage elements (Fig 4A), the same being true of the pectoral fin (Fig 4B). In adult scales, staining was observed in the edges of the scales only (Fig 4C).

### mmp-9

**Fig 5:**
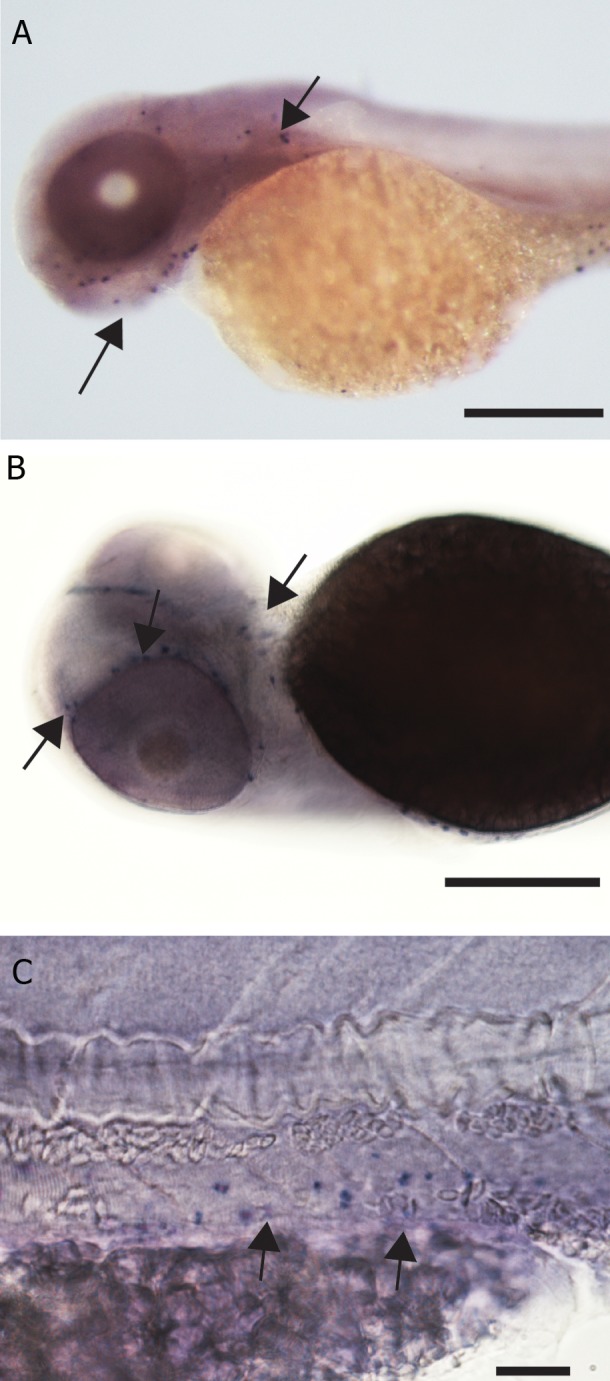
*mmp-9* gene expression in zebrafish embryos; A. 2 dpf embryo with numerous cells expressing *mmp-9* gene in the head region (arrows), scale bar=150 μm. B. 2 dpf embryo, ventral view, scale bar=100 μm. D. 4 dpf larva with expression in the ventral blood vessel in the posterior region of the body, scale bar=30 μm.

Hybridization patterns with *mmp-9* antisense probes are illustrated in fig 5. At 1 dpf, hybridization was seen in the ventral blood island (also the site of *rank* and *Ctsk* expression. Expression of *mmp-9* was also observed in the posterior extension of yolk sac. At 2 dpf, hybridization was seen in numerous, scattered cells in the head, pharyngeal region, on the yolk sac, near the ventral aorta and around the gut (Figes5A and B). At 3 dpf, there was hybridization in individual cells scattered in the tissue including pharyngeal region and caudal hematopoietic tissue (Fig 5C). At 4 and 5 dpf, a few cells expressing *mmp-9* were found scattered on the rostral part of the body, but were less numerous than at earlier stages.

We hybridized adult zebrafish scales as positive controls for *mmp-9* expression. There was expression in the radii of the scales in monoand multinucleated cells shown in our previous work (29) . Specific and strong expression was observed in the margins of the scales similar to that of *Ctsk* expression. Higher magnification shows expression in the multinucleated cells within the radii. There was no specific expression in the margins or radii of the scales hybridized with the *mmp-9* sense probe.

### TRAcP enzyme staining

**Fig 6:**
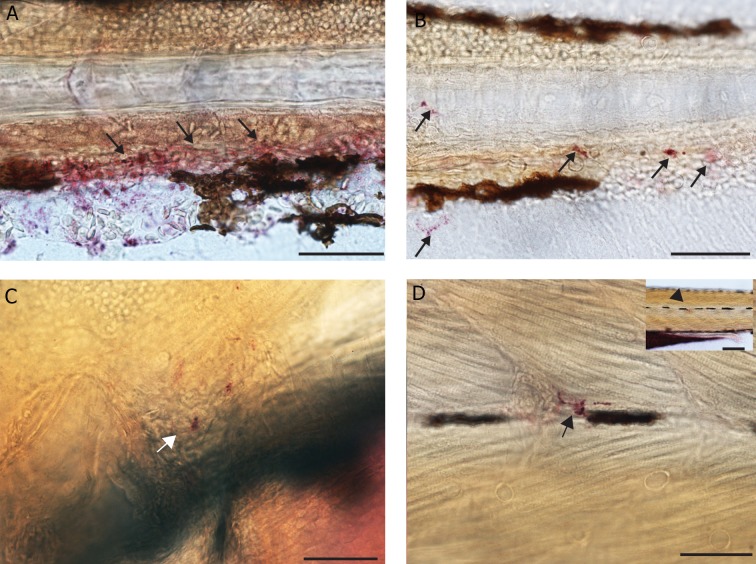
TRAcP histochemical staining. A. 3 dpf larva showing expression in the caudal hematopoietic tissue and blood cells (arrows), scale bar 50 μm. B. Tail region of 4 dpf larva with expression in individual cells (arrows), scale bar 50 μm. C. 5 dpf larva with expression at the pectoral fin (white arrow), scale bar=50 μm. D. 5 dpf larva expressing TRAcP in a single cell on the left flank of caudal body region (arrow) inset showing H at low magnification, scale bar 50=μm.

Immunohistochemical staining with TRAcP enzyme was done on whole mount zebrafish larvae (Fig 6A-D). In 1 dpf embryos, strong and specific enzyme staining was seen in cells in the ventral blood island. At 2 dpf, expression in the cells in the heart and pericardium was visible (data not shown). Stained cells were also present in the ventral fin fold and along the caudal haematopoietic tissue or hematopoietic tissue (CHT). Also, in 3 dpf embryos, in the cells scattered very sparsely over the body (Fig 6A, B). There were numerous cells stained with TRAcP enzyme in the tail region around the notochord. At 4 dpf, there was staining at the rostral end of Meckel’s cartilage, in the pectoral fin and in the pericardial region. In the posterior region of the body, stained cells were present in the ventral vein. Similar to 4 dpf, the expression in 5 dpf larvae was in the Meckels cartilage, pectoral fin (Fig 6C) and along the ventral blood vessels and near the somites (Fig 6D).

**Fig 7:**
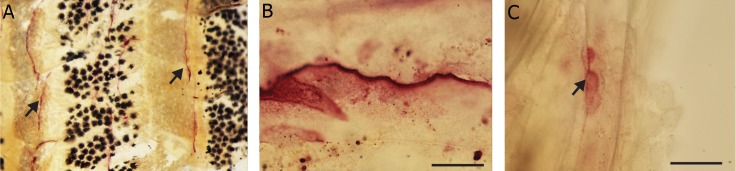
TRAcP histochemical staining. A. Scales on the skin of adult zebrafish expressing staining in the lateral margins (anterior to the top and dorsal to the right), scale bar=200 μm. B. Groove on a single scale with TRAcP staining, scale bar=20 μm. C. Putative multinucleated cell stained with TRAcP enzyme, scale bar=20 μm.

**Fig 8:**
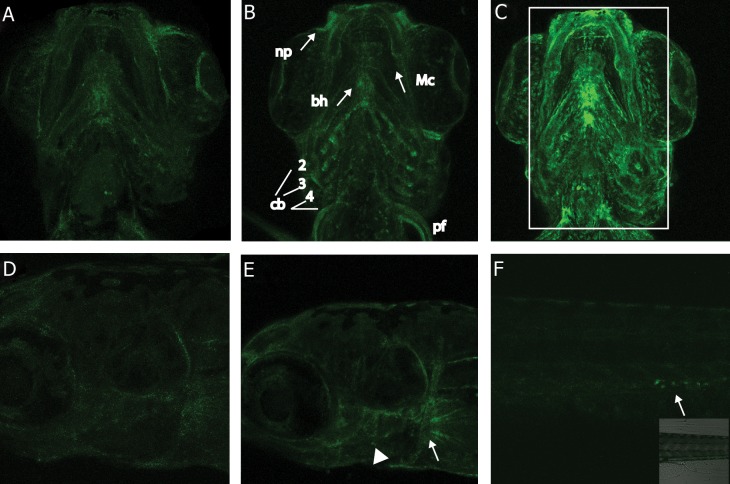
YFP labelled *Ctsk* transgenic zebrafish embryos. A. Confocal image showing 5 dpf larva with expression in pharyngeal skeleton (ventral view, anterior side upwards). B. DEX and vitaminutesD3 treated 5 dpf larva showing increased *Ctsk* transgenic expression in bh (basihyal), cb (ceratobranchials 2-5), Mc (Meckel’s cartilage), np (nasal placode), and pf (pectoral fin). C. DEX and vit D3 treated 5 dpf larva showing increased *Ctsk* expression, rectangle shows the area marked for fluorescence intensity assay. D. Confocal image showing left lateral view (anterior directed to the left) of the 5 dpf control larva with expression around ov (otic vesicle), cb (ceratobranchial), and pf (pectoral fin). E. Lateral view of DEX and vitaminutes D3 treated larva showing the skeletal elements with strong YFP signal, pharyngeal arches (arrow head) and pectoral girdle (arrow). F. Caudal region of a treated larva showing the ventral vein with scattered cells expressing YFP *Ctsk* (arrows).

TRAcP enzyme staining was also done on adult zebrafish scales as positive controls. Staining was observed at the lateral margins of the scales (Fig 8A) as well as very dense staining in some scales in the mid region along the grooves of normal scales (Fig 8B). However, no TRAcP staining was seen in the radii of the scales in our specimens. With the same staining multinucleated cells within the mid region of the normal scale were seen (Fig 8C).

### Transgenic *Ctsk* Larvae

YFP labelled *Ctsk* transgenic zebrafish larvae showed normal expression of *Ctsk* in the pharyngeal region (Fig 8A). These cells were more localised with the pharyngeal skeletal elements. Fluorescent expression was found around the pharyngeal arches, in the basihyal, Meckel’s cartilage, around the eye, around otic vesicle and in the pectoral girdle (Fig 8D).

**Fig 9:**
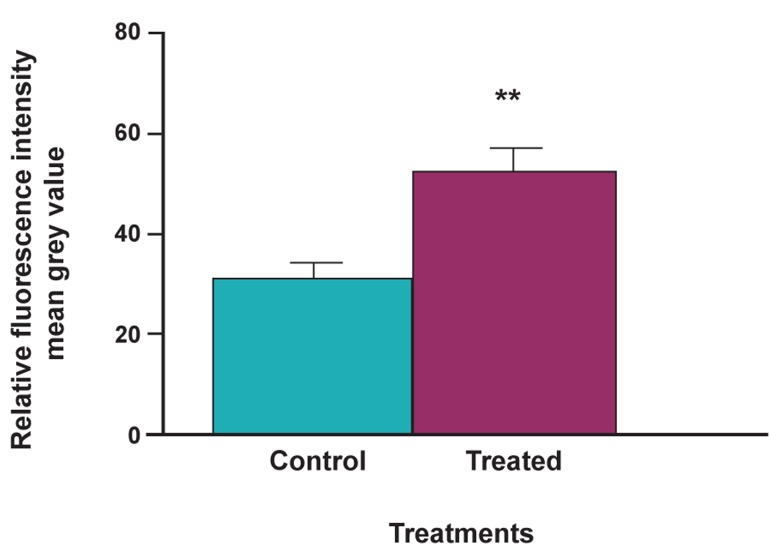
Measure of relative fluorescence intensity showing significant increase in case of DEX + vit D3. Lines represent standard error mean.

After exposing the embryos continuously with DEX and vitaminutes D3 for 4 days, the expression was more strongly seen around the pharyngeal skeleton, in np (nasal placode), Mc (Meckels Cartilage), Bh (basihyal), cb (ceratobranchial), and pf (pectoral fin) (Fig 8B, C, E). In the lateral view, *Ctsk* expression was clearly seen in the pharyngeal arches, ceratobranchials and the pectoral girdle (arrow) pf (pectoral fin), while there was also expression around the ov (otic vesicle). Although the intensity varied within the control and treated groups, when averaged over the individual embryos, was found to be significantly higher in the treatment group compared to controls, also noticeable in the images. The fluorescence intensity analysis also suggests a significant increase in *Ctsk* expression in the DEX + vitaminutes D3 treated larvae (Fig 9). However, a weaker *Ctsk* expression in the control transgenic larvae was observed (Fig 9). There was an interesting *Ctsk* expression in the scattered cells within the ventral vein near the caudal body region of the treated larvae similar to TRAcP and *mmp-9* (Fig 8F). These cells were also seen in the controls, but less frequent (Fig not shown).

## Discussion

We studied the expression in zebrafish embryos and larvae of a panel of genes that are associated with bone and tissue remodelling. Similarities in expression were evident between *Ctsk* and *rank*, on the one hand, and *mmp-9* and TRAcP on the other. *Ctsk* transgenic expression in normal embryos was associated with both type of expression as observed in *Ctsk* and *rank* as well as *mmp-9* and TRAcP.

### *Ctsk* and rank

In 1 dpf embryos, hybridized with *Ctsk* probe, adaxial staining was seen. We cannot say, however, whether this expression represents either the slow muscle precursors or sclerotomal elements which are known to occur in this region (30). *Ctsk* and *rank* expression was more restricted to the cells associated with pharyngeal skeleton and pectoral fin skeleton (31, 32) in embryos older than 1 dpf. It is known that by 5 dpf, osteogenesis occurs where cartilaginous skeleton transforms into calcified tissue (33). Also, this calcification or bone deposition occurs from outside in, that is the exterior part of bone is remodelled to deposit bone (34).

We found expression of *Ctsk* in the sections around pharyngeal arches, similar to the expression of SP7 in the perichondrium around the cartilage of a 4 dpf zebrafish embryos reported by DeLaurier et al. (34) Therefore, there are higher chances that this expression of *Ctsk* mRNA may be in the osteoclasts, around these tissues. It is also important to note here that we found strong similarities in the expression of *Ctsk* and *rank* genes in the larvae as well as in the adult scales. In the adult zebrafish scales, the *Ctsk* (35) and *rank* genes are both expressed in the marginal regions. This positive expression suggests that the genes which are known to carry osteoclasts are also expressed in the adult tissues (36). The scale-margin expression of *Ctsk* was found in multinucleated cells.

In addition, *Ctsk* reporter-line showed a similar pattern of *Ctsk* to the *in situ*s, with cells scattered along the pharyngeal arches. When the embryos were exposed at *prim-6* stage with 1 μM of DEX + vitaminutes D3 for 4 days and then observed with in vivo confocal microscopy, we found that there was much stronger expression of YFP *Ctsk* gene along the skeletal elements, as measured by fluorescent intensity. YFP expression was seen in all the skeletal elements which are known to have developed by this stage, compared to very weak expression in controls.

Our observations, therefore, suggest that there was activation in the YFP *Ctsk* expression in DEX and vitaminutes D3 treated embryos. It was also found that YFP *Ctsk* positive cells were present in the tail region along the ventral blood vessel. This expression in the ventral blood vessel is similar to the expression of TRAcP positive cells as well as *mmp-9* positive cells in normal untreated larvae. It can be, therefore, concluded that the *mmp-9* mRNA, TRAcP enzyme and YFP *Ctsk* protein expressing cells are not only found around the skeletal elements, but are also found scattered in other parts of body, specially blood vessels, which is logical considering hematopoetic origin of osteoclasts.

### *mmp-9* and TRAcP

In the present study, *mmp-9* and TRAcP enzyme were both expressed in cells sparsely scattered all over the body. Unlike *Ctsk* and *rank*, *mmp-9* and TRAcP expression was neither specifically associated with the pharyngeal arches nor the pectoral fin. Expression of *mmp-9* has been reported in cells scattered mostly on the head region and the posterio-lateral trunk, in embryos from 2-5 dpf (37, 38). The expression of *rank* in 1 dpf embryos in the ventral blood island and rostral blood island as well as expression of *mmp-9* in the ventral blood island are also interesting as this is the site of haematopoiesis in the early embryonic stages of zebrafish development (39).

TRAcP expression was observed in the skeletal structures such as Meckel’s cartilage only in 5 dpf larvae, but not in larvae younger than 5 dpf. Previous researchers have argued that TRAcP expression is a definitive marker for active osteoclasts (4). It is also worth considering here that the enzyme histochemistry does not seem to penetrate deep into the calcified tissue in whole mounts as much as YFP *Ctsk* expression. Further work is required to determine the nature of the cells expressing the markers characterized here.

In adult scales, the *mmp-9*, *Ctsk* and TRAcP expression is similar except for additional expression in the radii of the scales in the case of *mmp-9* hybridization. One possibility is that mononucleated cells expressing *mmp-9* in the radii of the scales are non-activated cells of the osteoclast lineage, whereas the multinucleated radial and marginal aggregates are mature osteoclasts. Nonetheless, the fact that marginal multinucleated cells in the adult scales express *mmp-9*, *Ctsk* and TRAcP which is suggestive of an osteoclastic lineage, as we found in other studies recently published for *mmp-9* and TRAcP expression in adult zebrafish scales (35, 36, 40). However, here, we for the first time present expression of *Ctsk* in the same cells and *RANK* in mononucleated cells.

## Conclusion

Our results show that genes associated with osteoclasts are expressed in early zebrafish development and in the multinucleated cells expressing *mmp-9* and *Ctsk* genes, and TRAcP enzyme, in adult scale osteoclasts. Our data with the transgenic *Ctsk* larvae suggests an association with the larval pharyngeal skeleton; this is also comparable to our expression data of *Ctsk* expression with *in situ* hybridisation. The TRAcP enzyme, *mmp-9* expression, and *Ctsk* YFP transgenic line also show expression in the blood cells found in the ventral vein. The expression of *Ctsk* in the reporter line was upregulated by DEX and vitaminutes D3 treatment. Together, these findings raise the possibility that osteoclast-like cells are present at early stages of zebrafish development, our functional studies also support this view.

## References

[1] Datta HK, Ng WF, Walker JA, Tuck SP, Varanasi SS (2008). The cell biology of bone metabolism. J Clin Pathol.

[2] Sims NA, Gooi JH (2008). Bone remodeling: Multiple cellular interactions required for coupling of bone formation and resorption. Semin Cell Dev Biol.

[3] Witten PE, Hansen A, Hall BK (2001). Features of mono- and multinucleated bone resorbing cells of the zebrafish Danio rerio and their contribution to skeletal development, remodeling, and growth. J Morphol.

[4] Witten PE, Huysseune A (2009). The unobtrusive majority: mononucleated bone resorbing cells in teleost fish and mammals. J Appl Ichthyol.

[5] Witten PE, Villwock W (2000). Bone resorption and bone remodelling in juvenile carp (Cyprinus carpio). J Appl Ichthyol.

[6] Witten PE, Holliday LS, Delling G, Hall BK (1999). Immunohistochemical identification of a vacuolar proton pump (VATPase) in bone-resorbing cells of an advanced teleost species, Oreochromis niloticus. J Fish Biol.

[7] Zapata A, Amemiya CT (2000). Phylogeny of lower vertebrates and their immunological structures. Curr Top Microbiol Immunol.

[8] Witten PE, Huysseune A (2009). A comparative view on mechanisms and functions of skeletal remodelling in teleost fish, with special emphasis on osteoclasts and their function. Biol Rev Camb Philos Soc.

[9] De Vrieze E, Sharif F, Metz JR, Flik G, Richardson MK (2011). M Matrix metalloproteinases in osteoclasts of ontogenetic and regenerating zebrafish scales. Bone.

[10] Jilka RL (2003). Biology of the basic multicellular unit and the pathophysiology of osteoporosis. Med Pediatr Oncol.

[11] Takeshita S, Kaji K, Kudo A (2000). Identification and characterization of the new osteoclast progenitor with macrophage phenotypes being able to differentiate into mature osteoclasts. J Bone Miner Res.

[12] Ibbotson KJ, Roodman GD, McManus LM, Mundy GR (1984). Identification and characterization of osteoclast-like cells and their progenitors in cultures of feline marrow mononuclear cells. J Cell Biol.

[13] Baron R, Neff L, Tran VP, Nefussi JR, Vignery A (1986). Kinetic and cytochemical identification of osteoclast precursors and their differentiation into multinucleated osteoclasts. Am J Pathol.

[14] Witten PE, Bendahmane M, bou-Haila A (1997). Enzyme histochemical characteristics of osteoblasts and mononucleated osteoclasts in a teleost fish with acellular bone (Oreochromis niloticus, Cichlidae). Cell Tissue Res.

[15] Ballanti P, Minisola S, Pacitti MT, Scarnecchia L, Rosso R, Mazzuoli GF (1997). Tartrate resistant acid phosphate activity as osteoclastic marker: sensitivity of cytochemical assessment and serum assay in comparison with standardized osteoclast histomorphometry. Osteoporos Int.

[16] Theill LE1, Boyle WJ, Penninger JM (2002). RANK-L and RANK: T cells, bone loss, and mammalian evolution. Annu Rev Immunol.

[17] Hsu H, Lacey DL, Dunstan CR, Solovyev I, Colombero A, Timms E (1999). Tumor necrosis factor receptor family member RANK mediates osteoclast differentiation and activation induced by osteoprotegerin ligand. Proc Natl Acad Sci USA.

[18] Nakashima T, Kobayashi Y, Yamasaki S, Kawakami A, Eguchi K, Sasaki H (2000). Protein expression and functional difference of membrane-bound and soluble receptor activator of NF-kappaB ligand: modulation of the expression by osteotropic factors and cytokines. Biochem Biophys Res Commun.

[19] O'Brien CA, Gubrij I, Lin SC, Saylors RL, Manolagas SC (1999). STAT3 activation in stromal/osteoblastic cells is required for induction of the receptor activator of NF-kappaB ligand and stimulation of osteoclastogenesis by gp130-utilizing cytokines or interleukin-1 but not 1,25-dihydroxyvitaminutesD3 or parathyroid hormone. J Biol Chem.

[20] Kurihara N, Chenu C, Miller M, Civin C, Roodman GD (1990). Identification of committed mononuclear precursors for osteoclast-like cells formed in long term human marrow cultures. Endocrinology.

[21] Udagawa N, Takahashi N, Akatsu T, Tanaka H, Sasaki T, Nishihara T (1990). Origin of osteoclasts: mature monocytes and macrophages are capable of differentiating into osteoclasts under a suitable microenvironment prepared by bone marrow-derived stromal cells. Proc Natl Acad Sci USA.

[22] Shuto T, Kukita T, Hirata M, Jimi E, Koga T (1994). Dexamethasone stimulates osteoclast-like cell formation by inhibiting granulocyte-macrophage colony-stimulating factor production in mouse bone marrow cultures. Endocrinology.

[23] Ram M, Sherer Y, Shoenfeld Y (2006). Matrix metalloproteinase- 9 and autoimmune diseases. J Clin Immunol.

[24] Kini RM, Zhang CY, Tan BK (1997). Pharmacological activity of the interdomain segment between metalloproteinase and disintegrin domains. Toxicon.

[25] Kini RM, Evans HJ (1992). Structural domains in venom proteins: evidence that metalloproteinases and nonenzymatic platelet aggregation inhibitors (disintegrins) from snake venoms are derived by proteolysis from a common precursor. Toxicon.

[26] Wilkinson DG (1998). In situ hybridization: a practical approach.

[27] Bussmann, Schulte-Merker S (2011). Rapid BAC selection for tol2-mediated transgenesis in zebrafish. Development.

[28] Kimmel CB, Ballard WW, Kimmel SR, Ullmann B, Schilling TF (1995). Stages of embryonic development of the zebrafish. Dev Dyn.

[29] de Vrieze E, Sharif F, Metz JR, Flik G, Richardson MK (2011). Matrix metalloproteinases in osteoclasts of ontogenetic and regenerating zebrafish scales. Bone.

[30] Germanguz I, Lev D, Waisman T, Kim CH, Gitelman I (2007). Four twist genes in zebrafish, four expression patterns. Dev Dyn.

[31] Flores MV, Lam EY, Crosier P, Crosier K (2006). A hierarchy of Runx transcription factors modulate the onset of chondrogenesis in craniofacial endochondral bones in zebrafish. Dev Dyn.

[32] Schilling TF, Kimmel CB (1997). Musculoskeletal patterning in the pharyngeal segments of the zebrafish embryo. Development.

[33] Du SJ, Frenkel V, Kindschi G, Zohar Y (2001). Visualizing normal and defective bone development in zebrafish embryos using the fluorescent chromophore calcein. Dev Biol.

[34] DeLaurier A1, Eames BF, Blanco-Sánchez B, Peng G, He X, Swartz ME (2010). Zebrafish sp7: EGFP: a transgenic for studying otic vesicle formation, skeletogenesis, and bone regeneration. Genesis.

[35] Azuma K, Kobayashi M, Nakamura M, Suzuki N, Yashima S, Iwamuro S (2007). Two osteoclastic markers expressed in multinucleate osteoclasts of goldfish scales. Biochem Biophy Res Commun.

[36] de Vrieze E1, Sharif F, Metz JR, Flik G, Richardson MK (2011). Matrix metalloproteinases in osteoclasts of ontogenetic and regenerating zebrafish scales. Bone.

[37] Volkman HE1, Pozos TC, Zheng J, Davis JM, Rawls JF, Ramakrishnan L (2010). T Tuberculous granuloma induction via interaction of a bacterial secreted protein with host epithelium. Science.

[38] Lewis RS, Stephenson SE, Ward AC (2006). Constitutive activation of zebrafish Stat5 expands hematopoietic cell populations in vivo. Exp Hematol.

[39] Warga RM, Kane DA, Ho RK (2009). Fate mapping embryonic blood in zebrafish: multi- and unipotential lineages are segregated at gastrulation. Dev Cell.

[40] Nemoto Y, Higuchi K, Baba O, Kudo A, Takano Y (2007). Multinucleate osteoclasts in medaka as evidence of active bone remodeling. Bone.

